# Case of Axillary Aneurism, in Which the Subclavian Artery Was Successfully Secured in a Ligature

**Published:** 1831-06

**Authors:** Valentine Mott


### AXILLARY ANEURISM.

### *Case of Axillary Aneurism, in which the Subclavian Artery was successfully secured in a Ligature.* By Valentine Mott, M.D.*

William Hines, aged twenty-eight, of Smithville, Virginia, came to New York, August 24th, 1830, and became my patient.

The account he gave of his case was, "that, about seven weeks ago, he received a violent strain while carrying a canoe on hand-bars across the arms, which was followed by an extensive discoloration of the skin of the right arm, extending to the chest, and attended with considerable pain: it, however, yielded to the usual remedies in such cases. Three weeks subsequent to the accident, he observed a swelling, about the size of a pigeon's egg, under the right arm, which had rapidly increased."

On examination, I found a tumor about the size of a goose's egg, and decidedly an aneurism of the axillary artery. His general health being good, I directed him to keep quiet, to be bled, and to take some purgative medicines; and fixed on Monday, the 30th, for tying the subclavian artery.

* American Journal of Med. Sciences.

At eleven o'clock A.M. he was placed upon the table, with the shoulders elevated and inclined to the right side. An oblique incision was made, two inches in length, through the integuments and platisma myoides muscle, and corresponding to a middle line of the triangular interval formed on the inner side by the scalenus muscle, on the outer by the omo-hyoideus, and below by the clavicle. The cervical fascia was next divided to the extent of an inch, and, with the fore-finger and the handle of a knife, the adipose and cellular tissues were put aside, and the artery readily exposed as it passes from between the scaleni muscles. After denuding the artery a little of the filamentous tissue, with a knife rounded at the point and cutting only at the extremity, a ligature was conveyed around it, from below upward, by the *American needle*, and the artery tied a little without the scaleni muscles.

No other ligature was required. The patient lost less than two teaspoonfuls of blood. The operation lasted about fifteen minutes, and was performed, with the assistance of Drs. Vache and Hosack, in the presence of Drs. Barrow, Kissam, Rogers, and Wilkes. The wound was closed by two stitches and adhesive straps; the arm was immediately wrapped in cotton wadding. No diminution of temperature took place.

Eight P.M. Found the patient comfortable: says he has less pain in the arm than before the operation; heat rather more than natural; a faint pulsation in the right radial artery; pulse eighty-eight.

31st, morning. Passed a comfortable night, after taking fifteen drops of the Sol. Sulph. Morphine, which was given to allay the pain about the elbow, and which he considered rheumatic, having had more or less of it for some time previous to the operation. This pain was no doubt caused by the pressure of the tumor upon the brachial plexus. Pulse seventy; skin natural; says that he feels very comfortable.

Evening. Complains of headach: directed a saline cathartic; pulse ninety; skin pleasantly moist; pulsation in the right radial artery occasionally very distinct and regular; temperature of the right arm a little higher than that of the left.

September 1st. Pain of the arm obliged him to sit up most of the night in an easy chair. After the operation of the salts, took again fifteen drops of the morphine, and slept quietly about five hours. Feels at present very comfortable; pulse seventy-five; not the least evidence of febrile disturbance in any of his symptoms.

2d. Feels much more comfortable than yesterday; slept composedly all night; little or no pain in the arm; pulse eighty. Removed the wadding from the arm, and enveloped it in flannel, which keeps it very comfortable.

3d. Slept well all night, after taking his dose of morphine, and feels very well today; pulse seventy-four; pulsation of the right radial more regular and distinct.

4th and 5th. Continues to improve.

6th and 7th. Every way comfortable; right radial pulsates regularly, theugh more feeble than the left.

9th. Dressed the wound, and removed the stitches: mostly healed, except where the ligature from the artery passes out. Pain in the arm for some days past has not been felt; makes no complaint; pulse in the radial artery very distinct and regular with the actions of the heart.

11th. Dressed the wound, which looks remarkably well; every thing appears very favorable.

14th. On removing the dressings today, the ligature came away; all promises well.

20th. Wound being just closed, permitted him to walk about the room, and to take his usual allowance of food. Aneurismal tumor much diminished in size, and very hard.

27th. Left the city today, on his return by water to Virginia.

When I reflect on the disease for which this operation was performed, and upon the situation, importance, and size of the vessel which was tied for its removal, it appears to me almost incredible that but twenty-seven days should have been required for its cure. That it should have succeeded, is particularly grateful to my feelings, inasmuch as it was first successfully performed by an American surgeon, (Dr. Post, of New York,) and is an additional proof of the triumph of surgery over disease and death.

